# Infectious hematopoietic necrosis virus (IHNV) persistence in Sockeye Salmon: influence on brain transcriptome and subsequent response to the viral mimic poly(I:C)

**DOI:** 10.1186/s12864-015-1759-y

**Published:** 2015-08-26

**Authors:** Anita Müller, Ben J. G. Sutherland, Ben F. Koop, Stewart C. Johnson, Kyle A. Garver

**Affiliations:** Fisheries and Oceans Canada, Pacific Biological Station, 3190 Hammond Bay Road, Nanaimo, V9T 6N7 British Columbia, Canada; Department of Biology, Centre for Biomedical Research, University of Victoria, Victoria, British Columbia, V8W 3N5 Canada; Present address: Département de biologie, Institut de Biologie Intégrative et des Systèmes (IBIS), Université Laval, Québec, G1V 0A6 Canada

**Keywords:** Sockeye Salmon, Infectious hematopoietic necrosis virus (IHNV), Brain, IHNV persistence, Poly(I:C) injection, Immune response, Transcriptomics

## Abstract

**Background:**

Sockeye Salmon are an iconic species widely distributed throughout the North Pacific. A devastating pathogen of Sockeye Salmon is infectious hematopoietic necrosis virus (IHNV, genus Novirhabdovirus, family *Rhabdoviridae*). It has been postulated that IHNV is maintained in salmon populations by persisting over the life of its host and/or by residing in natural reservoirs other than its susceptible hosts. Herein we demonstrate the presence of IHNV in the brain of Sockeye Salmon that survived an experimentally-induced outbreak, suggesting the presence of viral persistence in this susceptible species. To understand the viral persistent state in Sockeye Salmon we profiled the transcriptome to evaluate the host response in asymptomatic carriers and to determine what effects (if any) IHNV exposure may have on subsequent virus challenges.

**Results:**

A laboratory disease model to simulate a natural IHNV outbreak in Sockeye Salmon resulted in over a third of the population incurring acute IHN disease and mortality during the first four months after initial exposure. Nine months post IHNV exposure, despite the absence of disease and mortality, a small percentage (<4 %) of the surviving population contained IHNV in brain. Transcriptome analysis in brain of asymptomatic virus carriers and survivors without virus exhibited distinct transcriptional profiles in comparison to naïve fish. Characteristic for carriers was the up-regulation of genes involved in antibody production and antigen presentation. In both carriers and survivors a down-regulation of genes related to cholesterol biosynthesis, resembling an antiviral mechanism observed in higher vertebrates was revealed along with differences in nervous system development. Moreover, following challenge with poly(I:C), survivors and carriers displayed an elevated antiviral immune response in comparison to naïve fish.

**Conclusions:**

IHN virus persistence was identified in Sockeye Salmon where it elicited a unique brain transcriptome profile suggesting an ongoing adaptive immune response. IHNV carriers remained uncompromised in mounting efficient innate antiviral responses when exposed to a viral mimic. The capacity of IHNV to reside in asymptomatic hosts supports a virus carrier hypothesis and if proven infectious, could have significant epidemiological consequences towards maintaining and spreading IHNV among susceptible host populations.

**Electronic supplementary material:**

The online version of this article (doi:10.1186/s12864-015-1759-y) contains supplementary material, which is available to authorized users.

## Background

Sockeye Salmon (*Oncorhynchus nerka*), with their signature red spawning color, are an iconic species that is widely distributed throughout the North Pacific having a range that extends northwards from Oregon through Canada to Alaska, USA and from Hokkaido Island, Japan to Kamchatka Peninsula, Russia [1]. They are a key component of marine and freshwater ecosystems, and are highly valued as a food fish.

One of the most devastating pathogens of Sockeye Salmon is infectious hematopoietic necrosis virus (IHNV). The virus is enzootic throughout the range of Sockeye Salmon where it can also infect trout and other salmon species [2, 3]. IHNV is a negative-sense single-stranded, enveloped RNA virus assigned into the genus Novirhabdovirus within the family *Rhabdoviridae* [4]. First described in Sockeye Salmon in the early 1950s [5, 6], infection with this virus may cause an acute systemic disease, called infectious hematopoietic necrosis (IHN), with symptoms that may include lethargy, aberrant swimming, petechiae (pinpoint bleedings) and ascites (fluid in the peritoneal cavity) (reviewed in [7]). Outbreaks of IHN have predominately occurred in pre-smolt stages (alevin and fry) of Sockeye Salmon [8–11] although mortalities in smolts has been documented [12–14]. In adult Sockeye Salmon, the IHN virus is commonly found in spawning fish yet it is not associated with disease [15, 16].

Due to the significance of IHN in salmon populations, an extensive body of literature exists concerning the molecular characterization, pathogenesis, host response, and vaccination of this virus (reviewed in [7]). However, key questions regarding the epizootiology of IHNV remain unresolved, such as how IHNV is maintained in wild salmon populations. It has been suggested that adult salmon get re-infected with IHNV from a marine or freshwater source during their spawning migration, and/or that juveniles that survive virus exposure may become life-long asymptomatic carriers of IHNV, with the virus reactivating due to stress of the spawning migration [7].

Evidence in support of an asymptomatic IHNV carrier state has been reported [17] yet detection of IHNV carriers has not been consistent across studies possibly due to differences in the diagnostic methods used and the tissues examined [18, 19]. LaPatra et al. [19] demonstrated the presence of IHN virus in the brain of one out of 30 rainbow trout (*Oncorhynchus mykiss*) surviving the virus infection, however no virus was detected in kidney tissue. More recently, we have identified juvenile Sockeye Salmon in the marine environment that carry IHNV in their brains (*K.A. Garver and S.C. Johnson, unpublished*), however the consequences of these infections are unknown.

Although viral carrier states, often referred to as viral persistence, is well recognized and studied for a variety of viruses of mammals [20–24] it has received much less attention in lower vertebrate groups, including fish. Evidence of persistence of fish viruses has been found for infectious pancreatic necrosis virus (IPNV) [25], nodavirus [26], koi herpesvirus (KHV) [27] and viral hemorrhagic septicemia virus (VHSV) [28, 29]. However, knowledge of how viral persistence is developed and maintained is very limited, with some information available for nodavirus, IPNV and KHV [30–33]. Furthermore, with the exception of studies on nodavirus carriers in Atlantic cod (*Gadus morhua*) [32], studies examining the consequences of persistent viral infections in fish as they relate to the ability to respond to subsequent pathogen exposure have been limited.

Here we investigate the asymptomatic carrier state to improve our understanding of persistent IHNV infections in Sockeye Salmon populations, as well as explore the potential consequences of the carrier state on subsequent immune responses. Utilizing laboratory-controlled virus exposures we identify viral persistence in asymptomatic Sockeye Salmon nine months post challenge. We then profile transcriptome differences between brain tissue obtained from naïve (no virus exposure), survivors (exposed to IHNV but tested negative for IHNV in brain), and carriers (exposed to IHNV and tested positive for IHNV in brain). Additionally, for all groups a subset of individuals were stimulated with the viral mimic polyriboinosinic polyribocytidylic acid (poly(I:C)) to evaluate the effect of the carrier status on subsequent immune responses. These data are a first step in improving our understanding of how and under what circumstances persistent infection with IHNV occurs in Sockeye Salmon, and the potential consequences of such infections.

## Methods

### Fish source and husbandry

In June 2011 Sockeye Salmon fry (Sakinaw Lake stock, brood year 2010) were transferred from Rosewall Creek hatchery, Fanny Bay, British Columbia (B.C.) to the Pacific Biological Station (PBS), Nanaimo, B.C.. Fish were reared in 5 °C (±1 °C) dechlorinated freshwater under a natural photoperiod and fed dry pellets (EWOS) at 1 % body weight per day. The source hatchery for the fish used in this study has remained free of IHNV since its initiation of Sockeye Salmon culture in 1999 (*L. Clint, personal communication*) moreover viral screening a subset of 60 fish prior to transport to PBS, proved negative for the presence of IHNV using cell culture assay [34].

### Generation of IHNV survivors and carriers

**Fig. 1:**
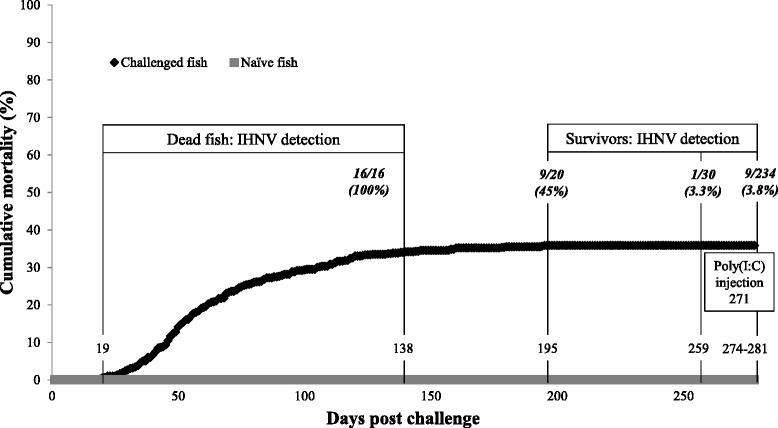
Cumulative mortality after IHNV challenge. Sockeye Salmon were exposed to waterborne IHNV (challenged fish) or left unhandled (naïve fish). Randomly selected dead fish sampled between 19 and 138 days post challenge (dpc) were tested for the presence of virus in the anterior kidney by cell culture assay. Brains of surviving fish were screened by RT-rPCR for the presence of IHNV at indicated time points. The percent of virus-positive fish is denoted in parentheses and is calculated from the number of positive detections out of the total number of fish tested (italicized numbers separated by a slash). The injection of poly(I:C) occurred at 271 dpc

In November 2011 Sockeye Salmon (600 fry, average weight = 5.5 g) were immersed for one hour in a 85 L static, aerated, 5 °C freshwater bath containing IHNV (Okanagan 2000 isolate, U genogroup; *K.A. Garver, unpublished*) at a dose of 1.98 × 10^3^ pfu ml^−1^. The isolate and dose of IHNV was selected based on past challenge work in Sockeye Salmon to initiate acute IHNV disease in the population (*K.A. Garver, unpublished*). After the one hour exposure to virus, the fish along with bath water were transferred into a 700 L tank. Sockeye Salmon from a separate tank that were not handled or exposed to IHNV served as control fish (naïve fish). Challenged and naïve fish were maintained in freshwater in one tank each, fed dry pellets (EWOS), and monitored daily for signs of disease and morbidity for a period of nine months. To mimic temperature changes that would be normally experienced in freshwater, fish were held for four months at 5 °C (±1 °C) followed by a slow transition over several days to 9 °C (±1 °C) for the remaining five months. At the end of the study (nine months post challenge), naïve and surviving Sockeye Salmon had similar average weights of 23 g and 20 g, respectively. All dead fish were individually bagged and frozen at -80 °C for use in diagnostic testing. Dead fish included moribund individuals that showed severe lethargy or lack of swimming that were consequently euthanized. To confirm presence of IHNV in mortalities during the acute infection phase, anterior kidney of 16 dead fish collected between 19 and 138 days post challenge (dpc) (Fig. 1) were examined using a cell culture plaque assay following methods in [35]. An IHNV RT-rPCR with specific primers to the virus nucleocapsid gene was used to evaluate viral persistence in survivors sampled at 195, 259, 274, 278 and 281 dpc. Use of research animals complied with Fisheries and Oceans Canada Pacific Region Animal Care Committee (AUP #11-024).

### Subsequent injection with polyriboinosinic polyribocytidylic acid (poly(I:C))

At 271 dpc, 240 fish from each of the challenged and naïve groups were reassigned into smaller 400 L tanks (120 fish per tank; total of four tanks) supplied with the same source of water. Shortly after the transfer, one group each of the challenged and naïve fish received an intraperitoneal (ip) injection of poly(I:C) (Sigma-Aldrich) dissolved in saline solution at a dose of 10 μg per g fish, thus the total amount of poly(I:C) was 200 and 230 μg for surviving and naive fish, respectively. For controls, a group each of challenged and naïve fish were transferred without injection. A sample of 39 fish were removed from each tank at 3 (274 dpc), 7 (278 dpc) and 10 (281 dpc) days after poly(I:C) injection/transfer. At sampling fish were euthanized in an overdose of buffered tricaine methanesulfonate (TMS) and brain and anterior kidney tissues were aseptically removed, individually flash-frozen in liquid N_2_ and transferred to −80 °C until RNA extraction.

### Total RNA extraction and purification

Samples of brain and anterior kidney were individually homogenized in 1 ml TRIzol® (Life Technologies™) using one stainless steel bead (5 mm, Qiagen) and a TissueLyser II (Retsch Inc., Qiagen). Tissues were processed for 2 min at 25 Hz at room temperature as per manufacturer’s instructions (Life Technologies™). Total RNA was extracted following the TRIzol® protocol using 1-bromo-3-chloro-propane in place of chloroform. An aliquot of each sample was taken and reserved for IHNV diagnostic testing (see below). The remaining sample was then purified by treatment with DNase I using the RNase-Free DNase Set (Qiagen) and subsequently column-purified using the RNeasy® MinElute® Cleanup kit (Qiagen) following manufacturer instructions. The RNA quantity and quality of unpurified or purified extracts were determined by spectrophotometry (NanoDrop-1000) and 1 % agarose gel electrophoresis, then stored at −80 °C.

### IHNV molecular diagnostic testing

Reverse transcriptase real-time PCR (RT-rPCR) was used to screen the surviving fish for the presence of IHNV using cycling conditions, probes, primers, and cut-off point as described in [36]. Briefly, 2 μg unpurified total RNA was reverse transcribed to cDNA using High Capacity cDNA reverse transcription kit (Applied Biosystems) with random primers. Five μl of undiluted cDNA was added to each RT-rPCR reaction in at least duplicate, and rPCR screening tests were repeated to confirm positives. For the purpose of this paper we refer to fish with detectable IHNV in one or both tissues as “carriers”, while “survivors” refers to fish in which IHNV was not detected. The unexposed control fish are referred to as “naïve”.

### Microarray preparation and data acquisition

Purified total RNA samples from brain were transcribed to cDNA in a randomized order. From each sample, 200 ng total RNA was reverse transcribed to cDNA and subsequently amplified to labeled cRNA using Low Input Quick Amp Labeling kits (v6.5; Agilent Technologies) as per manufacturer’s instructions. Experimental groups were labeled with Cy5-CTP (Perkin Elmer) and these consisted of naïve (*n* = 7), survivors (*n* = 7), and carriers (*n* = 5), as well as poly(I:C)-injected naïve (*n* = 7), poly(I:C)-injected survivors (*n* = 7), and poly(I:C)-injected carriers (*n* = 2). All fish were sampled 274 dpc (i.e. three days after poly(I:C) injection with the exception of two carriers that were collected 278 dpc. The smaller sample size for carriers was a result of the few individuals that survived the exposure that ended up carrying the virus.

A common reference design [37] was created by synthesizing cRNA samples as described above but with Cy3-labeled CTP for two individuals from each of the six conditions (total in reference = 12 samples). Equimolar quantities of each of the conditions were pooled to create the reference pool. Samples were hybridized to randomly assigned cGRASP 4×44K salmonid arrays with previously reported probe annotation [38–40] (Agilent Technologies, AMADID: 025055) according to the manufacturer’s instructions. Slides were washed using the stabilization solution (Agilent Technologies) to prevent ozone degradation of signal and were held in an environment that limited exposure to ozone. Slides were scanned on a ScanArray® Express (Perkin Elmer) at 5 μm resolution with constant PMT settings (Cy5:75; Cy3:80) that had been optimized to obtain 1 % of all spots saturated.

Images were quantified using Imagene 8.1 (Biodiscovery) which allowed for spots of poor quality to be flagged. Background correction was performed by subtracting the median of the background signal from the median of the foreground signal for each spot. The resulting files were then imported to GeneSpring GX 11.5.1 (Agilent Technologies) for normalization and analysis, and after all negative raw values being set to 1.0, the samples were normalized by an intensity-dependent Lowess normalization [41]. Data has been uploaded to GEO under accession GSE65241. Probes were removed from the analysis if they did not meet the following requirements in at least 65 % of the samples in any one condition: raw values ≥ 500 in both channels and no poor quality flags.

### Transcriptome analysis

**Fig. 2:**
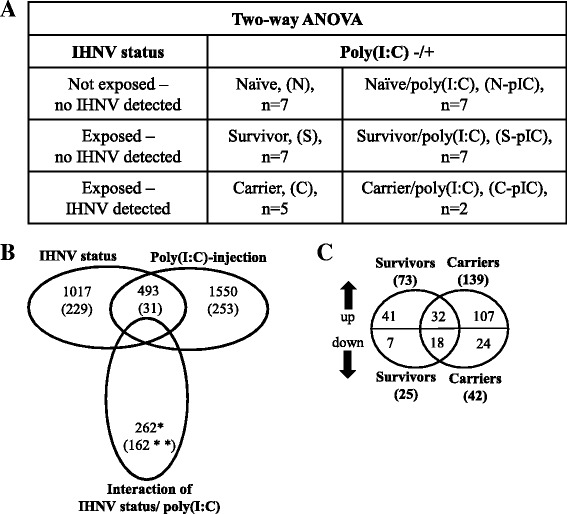
Overview of microarray analysis in brain of Sockeye Salmon. **a**) A two-way ANOVA (*p* ≤ 0.01) using expression data of the six groups naïve, survivors, carriers with and without poly(I:C) injection was performed. The IHNV status is indicated for each group. All samples (n) were collected 3 d post injection, i.e. 274 d post challenge (dpc), except for 2 carriers that were sampled 278 dpc. **b**) Venn diagram of probes affected by IHNV status, poly(I:C)-injection and interaction of both factors. Numbers in parentheses indicate numbers of probes retained after a fold change (FC) cut-off of ≥ 1.5. One asterisk (*) indicates that all interaction probes were removed from main effect lists to analyze separately. Two asterisks (**) refer to the number of probes used as input for *k*-means clustering shown in Fig. 3. **c**) Venn diagram of fold change-filtered up- and down-regulated probes affected by IHNV status that were separated into survivor- and carrier-relevant probes

A two-way ANOVA (*p* ≤ 0.01; no multiple test correction) was performed in GeneSpring (Agilent) to identify differentially expressed genes influenced by IHNV status (naïve, survivor, or carrier), poly(I:C)-injection (control or injected), or an interaction of both factors (Fig. 2). Probes with a significant interaction effect were removed from the main effect lists. Probes with a significant main effect of IHNV status were additionally filtered by fold change (FC), finding those probes with a 1.5-fold difference between survivor and control groups, or carrier and control groups concurrently in both poly(I:C) injected and non-injected groups. Genes specific to carriers or survivors were identified using a Venn diagram of up- or down-regulated genes (Fig. 2).

Probes with a significant main effect of poly(I:C) injection were additionally filtered by fold change to identify probes different between the poly(I:C)-injected and the non-injected control groups (*FC* ≥ 1.5) consistently in all of the following three comparisons: poly(I:C)-naïve vs. non-injected-naïve; poly(I:C)-survivor vs. non-injected-survivor; and poly(I:C)-carrier vs. non-injected-carrier.

Probes with a significant interaction between IHNV status and poly(I:C)-injection were filtered by fold change to retain probes that were differentially expressed by 1.5-fold between any two groups. These probes were then used as an input for a *k*-means clustering in GeneSpring (Agilent). Gene Ontology (GO) enrichment was performed with the GO FAT database in DAVID bioinformatics [42] using the Entrez-ID terms associated with the probes, and a background list of 14184 probes passing quality control filters (p-value cutoff). Venn diagrams were generated using Venny [43].

### Reverse transcriptase quantitative PCR

Reverse transcriptase quantitative PCR (RT-qPCR) was used to validate the microarray data. For each brain sample used in the microarray analysis, first-strand cDNA was synthesized from 400 ng of purified total RNA using the High Capacity RNA-to-cDNA™ Kit (Applied Biosystems) and 2.5 μM (final concentration) oligo d(T)_16_ primers (Applied Biosystems) according to the manufacturer’s instructions. Forward and reverse primers were designed in Primer3 [44] and their sequences are given in Additional file 1: Table S5.

Following cDNA synthesis, each 20 μl reaction was diluted 1:10 in nuclease-free water prior to use as template for RT-qPCR. Amplification was performed on a Mx3000P (Agilent) in a 20 μl reaction that consisted of diluted cDNA, 0.3 μM each of forward and reverse primer and 1X Power SYBR® Green PCR Master Mix (Applied Biosystem). The cycling program consisted of 1 cycle of 50 °C for 20 s, 1 cycle of 95 °C for 10 min and 40 cycles of 95 °C for 15 s and 60 °C for 1 min. For each sample the target transcript and reference gene were run in duplicates. PCR amplicons were tested for single products by melt curve analysis and sequenced on an ABI3130xL Genetic Analyzer (Life Technologies™) to confirm identities as per the manufacturer’s protocols using the RT-qPCR primers and a fluorescent dye terminator cycle sequencing kit (Applied Biosystems BigDye Terminator version 3.1). Primer efficiencies were determined using a serial dilution (2-fold, 5-point) of a pool of undiluted cDNA samples from all experimental conditions.

Relative expression levels were calculated by using the comparative Ct method for relative quantification including primer-specific efficiencies [45]. Naïve fish served as a calibrator and *U6 snRNA-associated Sm-like protein LSm8* (*LSM8*) and *dynein light chain 1 cytoplasmic* (*DYN*) were used as reference genes. The reference genes *LSM8* and *DYN* were selected due to their similar expression across samples on the microarray and in RT-qPCR and recent characterization as good reference genes for salmon [40]. To correlate results obtained by microarray and RT-qPCR analysis, linear best fit lines of log_2_ expression values for RT-qPCR samples versus microarray log_2_ expression ratios (Cy5/Cy3) were used for the probe corresponding to the contig used for primer design.

## Results

### Generation of IHNV survivors and carriers

A waterborne IHNV challenge was utilized to generate IHNV survivors and carriers. Mortality of IHNV-exposed Sockeye Salmon fry began at 19 dpc and continued until 195 dpc resulting in cumulative mortality of 35.3 % (Fig. 1). High mortality was observed during the initial four months following IHNV exposure (19–120 dpc) and accounted for over 92 % of the total mortality incurred during the experiment. After this four month period, mortality greatly subsided with only 17 mortalities occurring over the subsequent two and a half months (120–195 dpc). Virological analysis of a subset of 16 diseased fish collected between 19 and 138 dpc revealed the presence of IHNV with titers ranging from 1.35 × 10^3^ to 1.79 × 10^8^ pfu/g (median 1.77 × 10^7^ pfu/g). No mortality was observed in the naïve (unhandled) group.

With the exception of a few fish that developed spinal deformities such as scoliosis (sideways curvature) and/or lordosis (inward curvature), the majority of the Sockeye Salmon that survived appeared healthy and did not exhibit any signs of disease or distress at nine months post IHNV exposure. Using a highly sensitive IHNV RT-rPCR assay [36], we tested over 200 of these survivors for persistence of IHNV (Fig. 1). Although none of the survivors tested positive for IHNV in their anterior kidneys, IHNV was detected in the brains of 45 % (9/20), 3.3 % (1/30) and 3.8 % (9/234) of the fish examined at 195, 259, and 274/278/281 dpc, respectively. In these IHNV positive fish, the Ct-values ranged from 31 to 38. IHNV was not detected in brain or anterior kidney tissues of naïve fish (*n* = 10) sampled at 274 dpc that were used in the microarray study.

### Overview of microarray data

The response of Sockeye Salmon brain tissue to the presence of IHNV and/or poly(I:C) injection was determined using samples collected at 3 days after poly(I:C) injection (i.e. 274 dpc). Due to only three fish carrying IHNV at 274 dpc, two carriers (no poly(I:C) treatment) were also included from 278 dpc. This enabled the analysis of probes affected by IHNV status, by poly(I:C)-injection or by an interaction of the two factors (Fig. 2; Additional file 2: Table S1). With respect to probes that were identified as being responsive in the brain to IHNV exposure alone, a comparison of naïve fish to IHNV-exposed fish (i.e. carriers and survivors) identified more up-regulated probes than down-regulated: 73 and 139 probes were up-regulated among survivors and carriers, respectively, and only 25 and 42 probes were down-regulated. The majority (57 %) of these differentially expressed probes were specific to IHNV carriers while 21 % were specific to survivors, and 22 % were found in both carriers and survivors (Fig. 2). With respect to probes that were identified as being responsive in the brain to poly(I:C) injection, poly(I:C)-injection resulted in differential expression of 253 probes (203 up-regulated and 50 down-regulated) relative to the non-injected fish (Fig. 2). Notably, 31 probes had a significant main effect of both IHNV status and poly(I:C)-injection (Fig. 2). An interaction effect of both factors was found for 162 additional probes (Fig. 2). The following sections describe each of these types of responses. Complete lists of probes and GO enrichment analyses are shown in Additional files 3, 4 and 5 Tables S2, S3 and S4.

### Probes significantly affected by IHNV status

#### Immune response

GO analysis revealed an enrichment of adaptive immune responses in carriers relative to naïve fish (Additional file 3: Table S2). *Immunoglobulin-mediated immune response* (4 probes, *p* < 0.01) was enriched in IHNV carrier fish due to increased expression of *H-2 class II histocompatibility antigen gamma* chain (*CD74*), *complement C1q subcomponent subunit B* (*C1QB*) and *complement C1s subcomponent* (*C1S*) relative to naïve fish (Additional file 3: Table S2). Moreover, carrier-specific up-regulation of numerous *immunoglobulin* (*Ig*) probes for various regions of the light (*n* = 14) and heavy (*n* = 4) chains (FC from 1.9 to 9.8 (Table 1, Additional file 3: Table S2)) suggests an ongoing adaptive immune response in carriers. *Antigen processing and presentation of peptide antigen* (3 probes, *p* < 0.01) was significantly enriched by the carrier-specific up-regulation of *CD74* and *tapasin* (*TAPBP*). Also involved in this biological process are *cathepsin S* (*CTSS*), *beta-2-microglobulin* (*B2M*) as well as *proteasome subunit beta type-*6 (*PSMB6*) and *PSMB7* that were up-regulated in carriers relative to naïve fish. A third enriched immune-relevant biological process was *T cell differentiation* (3 probes, *p* < 0.05), but only two unique annotations, *CD74* and *coronin-1A* (*CORO1A*), were associated with this function.

**Table 1:** Fold changes of selected genes affected by IHNV status

Survivor- or carrier-relevant genes were obtained if fold changes were ≥1.5 between survivor (S) and naïve (N) fish or carrier (C) and naïve (N) fish concurrently in both poly(I:C)-injected (pIC) and non-injected groups. The gene *galectin-3-binding protein* marked with one asterisk (*) has been described as virus responsive gene (VRG) in Krasnov et al. [54]. Genes labeled with two asterisks (**) were also affected by main factor poly(I:C) injection (see Table 2). Colors refer to ranges of fold changes: yellow 1.5 to 2.5; orange 2.6 to 3.5; brown 3.6 to 9.8. Light green −2.5 to −1.5; green −3.1 to −2.6

In contrast, survivors did not have any probes or GO enrichment associated with antibody production, antigen presentation or T cell differentiation, with the exception of a weak up-regulation of two probes representing *Ig heavy chains* (*FC* = 1.6 to 1.9) and probe *117849 pfam09307* (*FC* = 1.7 to 2.2) that may interact with major histocompatibility complex (MHC) class II molecules to facilitate antigen presentation.

Relative to naïve fish, both carriers and survivors differentially expressed genes involved in inflammation. While carrier-specific probes included *transient receptor potential cation channel subfamily V member 1* (*TRPV1*), *C1QB*, *C1S* and *protachykinin-1* (*TAC1*), survivor-specific probes were *annexin A5* (*ANXA5*), *CCAAT/enhancer-binding protein delta* (*CEBPD*) and *peroxiredoxin* (*PRDX*). While carrier-specific probes contributed to the enrichment of the biological process *inflammatory response* (4 probes, *p* < 0.05), no GO enrichment was identified among survivor-specific probes. A few probes involved in innate immune responses, including inflammation showed the same patterns of expression in both carriers and survivors relative to naïve fish. These include *galectin-3-binding protein* (*LGALS3BP*) and *lysozyme g* (*LYG*) which were up-regulated and *interleukin-1 receptor-like 1* (*IL1R1*) and *latent-transforming growth factor beta-binding protein-1* (*LTBP1*) which were down-regulated. While these results indicate that innate immune responses occur in both carriers and survivors, adaptive immune responses take place only in carriers.

#### Lipid metabolism

Cholesterol is an essential component of cellular membranes and regulation of cholesterol synthesis may interfere with viral lifecycles [46]. In this study we identified multiple genes involved in cholesterol biosynthesis [47, 48] that were significantly down-regulated in carriers and survivors when compared to naïve fish (Table 1, Additional file 3: Table S2). These included *hydroxymethylglutaryl-CoA synthase cytoplasmic* (*HMGCS1*) and *farnesyl pyrophosphate synthetase* (*FDPS*) in both survivors and carriers, *diphosphomevalonate decarboxylase* (*MVD*) in survivors, and *sterol-4-alpha-carboxylate 3-dehydrogenase*, *decarboxylating* (*NSDHL*) only in carriers.

Genes with other roles in lipid metabolism had increased levels of expression in the brains of carriers and survivors when compared to naïve fish. Three carrier-specific probes (*FC* ≤ 2.4) included *GPI inositol-deacylase* (*PGAP1*), *phospholipase A1 member A* (*PLA1A*) and *proactivator polypeptide* (*PSAP*). *Lipoprotein lipase* (*LPL*) showed increased expression in both survivors (*FC* = 1.6) and carriers (*FC* = 1.5).

#### Nervous system development and function

Genes involved in the development and function of the nervous system were also affected by IHNV exposure. Twenty probes associated with this process were significantly up-regulated in carriers relative to naive fish, whereas only 6 probes (5 up- and 1 down-regulated) showed differentially expression in survivors compared to naïve fish (Table 1, Additional file 3: Table S2). GO enrichment analysis identified biological processes such as *forebrain development* (4 probes, *p* < 0.01) and *behavior* (5 probes, *p* < 0.05) only among carrier-specific up-regulated probes, while no biological processes were enriched among survivor-specific probes probably due to the low number of probes in this group.

Both carriers and survivors showed significant up-regulation of *basic leucine zipper and W2 domain-containing protein 2* (*BZW2*) which is possibly involved in neuronal differentiation, as well as *ependymin* (*EPD*) that may play a role in consolidation of memory and regeneration of neurons. The probes with highest fold changes related to nervous system function for both survivors and carriers were *pro-melanin-concentrating hormone* (*PMCH*) *1* and *2* (*FC* = 2.7 to 4.7 (survivors) and 3.9 to 7.2 (carriers)), a neuropeptide regulating body color and appetite in fish [49, 50]. Specific to survivors was the down-regulation of *myelin P0 protein* (*MPZ*), which is a component of myelin sheaths, the insulators of axons that facilitate efficient action potential conduction. These results suggest that the brain’s function may be affected in both carriers and survivors.

### Probes affected by poly(I:C)-injection

To determine whether a previous exposure to IHNV affects how Sockeye respond to other immunological challenges, both naïve and surviving Sockeye (survivors and carriers) were intraperitoneally injected with the viral mimic poly(I:C) and sampled 3 dpi. Two hundred and fifty three probes were identified that were affected by poly(I:C) regardless of the IHNV status (i.e. main effect of poly(I:C), no significant interaction effect) (Fig. 2). Enriched among the 203 up-regulated probes were GO categories *response to virus* (*p* < 0.01), *antigen processing and presentation* (*p* < 0.01), *immune response* (*p* < 0.01), *defense response* (*p* < 0.01) and *cellular protein catabolic process* (*p* < 0.01) suggesting a characteristic antiviral immune response in poly(I:C)-injected compared to non-injected fish regardless of IHNV status (Additional file 4: Table S3).

Viral RNA and poly(I:C) are detected by transmembrane and cytosolic pattern recognition receptors (PRR) [51]. Expression of *probable ATP-dependent RNA helicase DDX58* (*RIG-I*), a cytosolic PRR, was significantly up-regulated in poly(I:C)-injected Sockeye Salmon (Table 2, Additional file 4: Table S3). Activation of *RIG-I* may induce the production of type I interferon (IFN) mediated by signal transducers such as IFN regulatory factor (IRF) 3 and IRF7 [51]. In the poly(I:C)-injected Sockeye Salmon, *IRF3* and *IRF7* were also up-regulated, as were several type I IFN-stimulated genes including *IFN-induced GTP-binding protein Mx*, *IFN-induced protein 44*, *IFN-induced protein with tetratricopeptide repeats 5*, *IFN-induced very large GTPase1* and *radical S-adenosyl methionine domain-containing protein 2* (*Vig1*). Cellular responses to type I IFN are mediated by the Janus kinase (JAK) and signal transducer and activator of transcription (STAT) (JAK/STAT) signaling pathway [52, 53]. Genes coding for components of this pathway such as *STAT1* and *STAT1-alpha/beta* were up-regulated, indicating a role of this pathway in response to the injection of poly(I:C). Other up-regulated probes identified in poly(I:C)-injected fish were *sacsin* (*SACS*) and *galectin-9* that are commonly reported as up-regulated after poly(I:C) or virus treatment [54].

**Table 2:** Fold changes of selected genes affected by poly(I:C) injection

A probe was retained if fold changes were ≥1.5 in all three comparisons: poly(I:C)-injected naïve fish (N-pIC), survivors (S-pIC) or carriers (C-pIC) versus the respective non-injected group (N, S, C). Genes marked with (*) have been described as virus responsive genes (VRG) in Krasnov et al [54]. Genes labeled with (**) were also affected by main factor IHNV status (see Table 1). Colors refer to ranges of fold changes: yellow 1.5 to 2.5; orange 2.6 to 3.5; brown 3.6 to 10.0; red 10.1 and higher. Light green -2.5 to -1.5; green -3.5 to -2.6; dark green -3.6 and lower.

Other genes associated with immunity but not restricted to the innate antiviral response were also induced in poly(I:C)-injected fish (Table 2, Additional file 4: Table S3). These include genes involved in antigen processing and presentation such as *antigen peptide transporter 1* and *2* (*TAP1*, *TAP2*), *proteasome subunit beta type-8* (*PSMB8*) and *TAPBP*. Cell chemotaxis was indicated by the up-regulation of two probes annotated with *29114 cd01119, Chemokine_CC_DCCL*. There was also up-regulation of a probe annotated as *nicotinamide phosphoribosyltransferase* (*NAMPT*). This gene encodes a protein known to be involved in nicotinamide adenine dinucleotide (NAD) biosynthesis, but cytokine-like activity has also been reported for this protein (reviewed in [55]). Some of the highest fold changes (FC between 22.3 and 83.2) were found for probes annotated as *ubiquitin*. The functions of ubiquitin include, but are not limited to protein degradation, cell cycle, toll-like receptor (TLR) signaling, apoptosis and mRNA metabolism.

Also noteworthy is the up-regulation of several probes that are annotated to non-immune related genes that have been identified as viral responsive genes (VRG) by Krasnov et al. [54]. These include *deoxycytidine kinase* (*DCK*) that is required for nucleotide biosynthesis, and *poly [ADP-ribose] polymerase 12* (*PARP12*) that is involved in protein modification. In addition, the VRG *receptor-transporting protein 3* (*RTP3*) showed strong up-regulation (between 27.5 and 59.2) in poly(I:C)-treated Sockeye Salmon, though its function in response to viruses is mostly unknown [54].

Poly(I:C)-injection also resulted in down-regulation of 50 probes (Table 2, Additional file 4: Table S3). These include probes associated with innate immune responses including *complement component C7* (*C7*), *ANXA5*, *complement receptor type 2* (*CR2*) and *lysozyme C II*.

Common to both IHNV status and poly(I:C) injection (main effect lists) were 31 probes with 15 unique annotations (Additional files 3 and 4: Tables S2, S3). Within this list were 11 genes that were up-regulated in survivors and/or carriers, and down-regulated by poly(I:C)-injection. These include *beta-1,3-glucosyltransferase* (*B3GALTL*), *PRDX*, *plasma retinol-binding protein 1* (*RCP4A*), *EPD*, *ependymin-2* (*EPD2*) and *serotransferrin-2* (*TF2-11*). The remaining four genes (*40S ribosomal protein S2* (*RPS2*), *proteasome subunit beta type-7* (*PSMB7*), *TAPBP*, *LGALS3BP*) were up-regulated in survivors and/or carriers, and also up-regulated in poly(I:C)-injected fish, suggesting a general role of these genes in host response to foreign RNA.

### Probes affected by interaction of IHNV status and poly(I:C) injection

**Fig. 3:**
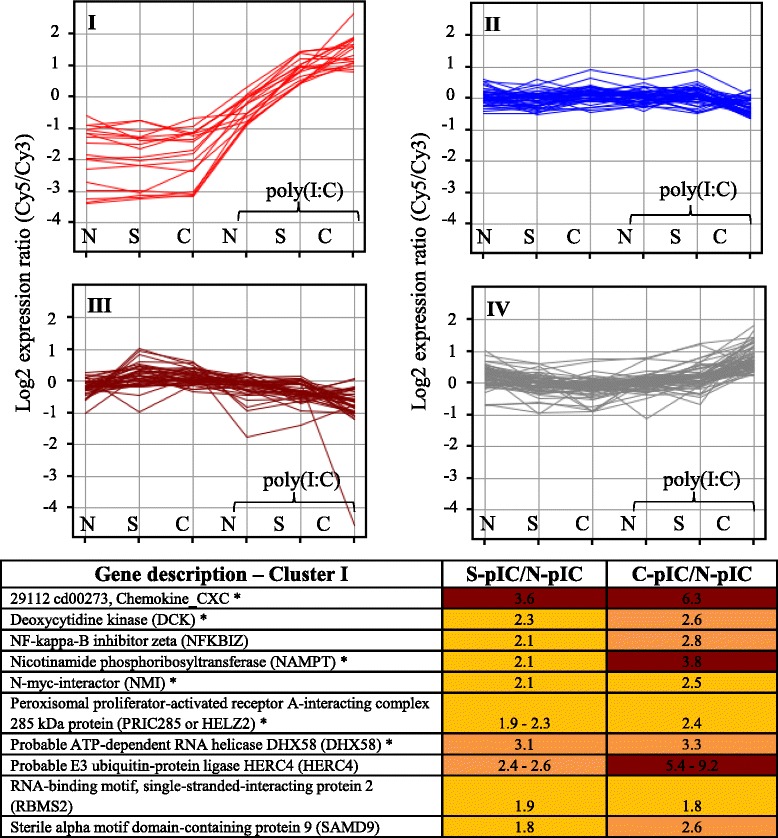
Cluster analysis of probes affected by interaction of poly(I:C)-injection and IHNV status. Based on expression levels of individual probes, clusters I to IV were generated containing 18, 45, 41 and 58 probes, respectively. Fold changes of uniquely annotated probes from cluster I are shown for poly(I:C)-injected survivors (S-pIC) and carriers (C-pIC) relative to naïve-injected fish (N-pIC) (fish group abbreviations as defined in Fig. 2). Colors refer to ranges of fold changes: yellow 1.5 to 2.5; orange 2.6 to 3.5; brown 3.6 to 9.2. Genes marked with an asterisk (*) have been described as virus responsive genes (VRG) in Krasnov et al. [54]

Some probes responded to poly(I:C) differently depending on the IHNV status of the group. These 162 probes were clustered by expression level for improved characterization to generate four distinct clusters (I to IV) with 18, 45, 41 and 58 probes, respectively (Fig. 3, Additional file 5: Table S4).

Probes in cluster I had similar expression among non-injected groups and up-regulation following poly(I:C)-injection, with highest expression in survivors and carriers. For some probes the highest expression was in injected carriers (Fig. 3, Additional file 5: Table S4). Although there were only ten uniquely annotated probes within this cluster, this cluster was enriched for genes involved in *defense response* (3 probes, *p* < 0.01) (Additional file 5: Table S4), including *N-myc-interactor* (*NMI*) that augments cytokine-mediated STAT transcription, *NF-kappa-B inhibitor zeta* (*NFKBIZ*) that is an inhibitor of NF-kappa-B transcription factors which play critical roles in inflammation, immunity, cell proliferation, differentiation and survival, and *probable ATP-dependent RNA helicase DHX58* (*DHX58*) which is another cytosolic PRR to detect foreign RNA. This cluster also contained *sterile alpha motif domain-containing protein 9* (*SAMD9*) that is involved in inflammatory responses to tissue injury, and *CXC chemokine* that may have multiple functions and may be involved in immune and homeostatic functions. Within this cluster, fold changes of most probes were similar in poly(I:C)-injected survivors and poly(I:C)-injected carriers relative to the poly(I:C)-injected naïve fish. An exception included probes (*n* = 4) annotated as *probable E3 ubiquitin-protein ligase HERC4* that is involved in modification and degradation of proteins, for which injected survivors and carriers were 2.4 to 2.6 fold and 5.4 to 9.2 fold, respectively, up-regulated relative to injected naïve fish (Fig. 3, Additional file 5: Table S4).

Cluster II was characterized by probes that showed minor changes (*FC* < 2) in expression between naïve, survivors and carriers prior to and following poly(I:C)-injection (Fig. 3; Additional file 5: Table S4). Cluster III included probes that varied in expression among non-injected groups, and had a slight decreased expression in injected groups (Fig. 3, Additional file 5: Table S4). In this cluster, injected survivors were similar to injected naïve fish, but several probes had lowest expression in injected carriers (*FC* ≥ 2 relative to injected naïve fish). These included probes with annotation to *GTP-binding protein 10*, involved in ribosome biogenesis, *cell division protein kinase 4*, involved in cell proliferation and *prostaglandin E synthase 3*, associated with inflammation and lipid metabolism. One probe with the largest down-regulation was *nuclease-sensitive element-binding protein 1* (*YBX1*) (*FC* = 23.4), which is involved in mRNA processing. Cluster IV contained probes with similar expression in non-injected fish. In injected fish, both carriers and survivors showed small but significant increases in expression (Fig. 3, Additional file 5: Table S4) and probes in injected naïve fish remained similar to those in non-injected fish. Within this cluster, enriched biological processes (*p* < 0.05) included metabolic processes (*polysaccharide metabolic process* (3 probes), *sulfur metabolic process* (3 probes), *chondroitin sulfate metabolic process* (2 probes) and *regulation of RNA metabolic process* (7 probes)) and *negative regulation of cell differentiation* (3 probes) (Additional file 5: Table S4).

### Validation of the microarray results by RT-qPCR

To validate data obtained by microarray analysis RT-qPCR was performed on 12 transcripts that were differentially expressed in the microarray analysis. These included *MX*, *SACS*, *LGALS3BP*, *immunoglobulin μ heavy chain (Ig HCmu)*, *EPD2*, *CEBPD*, *RTP3*, *C7*, *C1S*, *squalene monooxygenase* (*SQLE)*, *FDPS* and *B3GALTL* (see Additional file 1: Table S5 for full gene names). Log_2_ expression ratios (Cy5/Cy3) of the microarray vs. log_2_ expression values of the RT-qPCR analysis were correlated for each gene and individual as shown for *MX* in Additional file 6: Figure S1a. R^2^ and slope values from the best fit lines of these correlations are shown in Additional file 6: Figure S1b. The median R^2^ is 0.7. These results indicate that expression levels of the studied transcripts correlated well between the microarray and RT-qPCR analysis. An exception was *B3GALTL* whose expression change on the array could not be confirmed by RT-qPCR as indicated by its low R^2^ value of 0.2.

It was difficult to determine whether both secreted and membrane-bound forms of *Ig mu chain C region* were present on the array. Therefore we used primers specifically designed to amplify either the secreted or the membrane form of the *Ig mu chain* [56] by RT-qPCR. The results indicated that both the membrane-bound and secreted forms of *Ig mu chain* were specifically up-regulated in carriers. The probes best matching each of these sets of primers were highly correlated with their expression profiled by RT-qPCR (membrane-bound probe C078R010, secreted probe C017R015; both R^2^ = 0.9).

## Discussion

### Generation of IHNV survivors and carriers

Infectious hematopoietic necrosis virus is distributed widely on the Pacific Coast of Canada and the United States where species such as Sockeye Salmon are commonly infected. Despite the relatively frequent occurrence of IHNV in salmon and trout, our understanding of viral persistence after infection of these species remains uncertain. Here we utilized a laboratory disease model to simulate a natural IHNV outbreak in Sockeye Salmon with the aim of better defining the relationship between Sockeye Salmon and IHNV, whether the virus persists within this host, and if so what are the consequences of such infections.

To best simulate a natural virus infection, Sockeye Salmon were waterborne exposed to an IHNV strain that naturally occurs in Sockeye Salmon in British Columbia [3]. Additionally, virus exposure was conducted under a similar photoperiod and water temperature regime as experienced by the Sockeye Salmon in their natural rearing environment. Our challenge initiated an IHNV disease epizootic that resulted in 35.3 % cumulative mortality with surviving fish remaining free of any signs of disease. However, among a small number of these survivors, spinal deformities such as scoliosis and lordosis were observed as has been reported in other studies of IHNV surviving salmonids [6, 19]. Although it was possible to maintain these fish in a laboratory setting, it is extremely likely that in a natural setting these individuals would be quickly removed from the population due to their inability to avoid predation. Consequently because these fish are likely not sustained in the wild they were excluded from our analyses.

The asymptomatic survivors of infection were screened for the presence of IHNV in the brain and anterior kidney and although kidney tissue typically contains high viral loads during the acute stages of IHN disease [57], no virus was detected in this tissue of surviving fish. Conversely, IHNV was detected in brain tissue among these survivors, demonstrating that tissues of the central nervous system play an important role in IHNV persistence and substantiates its neurotropic potential [58]. Interestingly, the proportion of fish testing positive for IHNV in brain decreased from a high of 45 % immediately following the cessation of morbidity (195 dpc) to < 3.8 % at the time of the initiation of the microarray study (274 and 278 dpc). This declining viral persistence over time may be an indicator of those individuals in the population which either got infected later and/or simply retain virus longer than the majority of the surviving population.

McGavern and Kang, [59] described three possible outcomes of viral infection of the nervous system, all of which result in a host defense response. These are acute replication, persistence and latency. They define persistent viral infections as infections in which the virus undergoes continuous viral replication while latent infections are defined as a “dormant” state during which replication is minimized or ceased. With respect to IHNV, it is unclear what category the asymptomatic carriers observed in our study would fall into. Based on their transcriptome profiles and lack of virus in anterior kidney, it is unlikely that the IHNV carrier fish represents an acute replication stage. Typically during acute IHNV infections, when high titers of virus are present systemically, fish exhibit a strong antiviral immune response which can include up-regulation of *IRF1*, *IRF7*, *IFN-induced protein 44*, *IL-1β* and *Mx* [60, 61]. However in the asymptomatic carriers examined herein, such antiviral responses were not present suggesting that viral replication is limited. As the prevalence of IHNV positive fish decreased over time, it is possible that carriers simply represent an earlier stage of recovery that would eventually result in virus clearance or in reduced viral loads that are below detectable levels. While clearly more work needs to be done to define the infection status of those fish, in this study they are referred to carriers or persistently infected fish which is in accordance to the definition by Kane and Golovkina [24]. Similar reductions in virus load have been observed for other fish viruses such as KHV, nodavirus and VHSV [29, 62–64] and sometimes the occurrence of viral persistence is only made evident via a stress induced event that likely impairs host control [63]. Whether persistent IHNV in Sockeye Salmon observed herein could be reactivated and/or disrupt host control is an important avenue for further investigation.

### Gene expression influenced by IHNV status

To explore the differences in gene expression between those fish carrying IHNV versus those without detectable virus, we used the cGRASP 4x44K salmonid arrays recently validated in *Salmo salar* and *Oncorhynchus* spp. [40, 65], to measure whole brain transcriptional differences between these IHNV states. One of the main differences observed between asymptomatic survivors and carriers, is in their regulation of genes involved with antibody production. The importance of an antibody response in protecting salmon against IHNV has been well demonstrated by passive immunization studies using sera with neutralizing antibodies [66]. Several genes involved in antibody production were significantly up-regulated in the brains of carriers relative to naive fish. Interestingly, with exception of two weakly up-regulated probes, none of these genes were found to be differentially expressed when survivors were compared to naïve fish. As the whole brain tissue was examined it is uncertain if these genes were being expressed by peripheral leukocytes that had become associated with and/or entered the brain, expressed by cells of the central nervous system (CNS), or both. Peripheral leukocytes can be recruited to, and in some cases enter and persist within, the brain (reviewed in [67]). In response to virus infection, IgM+ lymphocytes have been reported to infiltrate the brain of gilthead seabream that was an asymptomatic carrier of viral nervous necrosis virus (VNNV) [31]. This observation led these authors to propose the major role of local adaptive immunity in the control of VNNV in this species. Whether the adaptive immune response in Sockeye Salmon is playing a similar role in IHNV carriers is uncertain.

Antibodies may neutralize or opsonize a pathogen, but may also be involved in activation of the complement system. The involvement of complement in IHNV carriers was indicated by the up-regulation of *C1QB* and *C1S* in carriers vs. naïve fish. These complement components bind antibodies that are part of an antibody-pathogen complex to initiate the complement cascade of the classical pathway. Noteworthy, antibodies may prevent spread of virus and infection of cells, but elimination of virus in already infected cells is probably dependent on cellular immunity involving cytotoxic cells. The GO enrichment identified *T cell differentiation* among carrier-relevant probes indicating a role of cellular immunity, although only three probes representing two known genes were associated with this function.

Alternatively, the expression of *Ig* in cells of the CNS in fish may be involved in neuroprotection and/or repair as seen in higher vertebrates. It has been recently demonstrated that immunoglobulin expression as mRNA and/or proteins can occur in the cells of CNS of murines and humans [68–70]. From these studies it appears that immunoglobulins participate in other non-immune related roles within the CNS. As an example, IgG gene and protein expression was found to be up-regulated in primary cultures of rat neurons in response to injury due to complement and microglial activation, suggesting a protective role of IgG against such injuries [70]. Whether the expression of genes related to antibody production indicate that carriers are in an earlier stage of the infection process, or whether this expression is related to neuroprotection remains unknown. In any case, a hallmark of IHNV carriers was a significant increased expression of *Ig* and *complement system components*.

Another function that was likely altered as a result of being an IHNV carrier is antigen presentation. Up-regulation of probes signaling an involvement of MHC class I pathways were evident in carriers. The probe *B2M* was a predominate indicator of the involvement of the MHC class I pathway although other probes involved in protein processing and peptide loading (i.e. *PSMB6*, *PSMB7* and *tapasin*) were also up-regulated among carriers. Overall the involvement of the MHC class I pathway being exclusive to carriers is parsimonious with the presence of IHNV in brains of these fish, as a main function of the MHC class I molecules is the presentation of intracellular antigens.

An involvement of professional antigen presenting cells (APCs) in response to IHNV was indicated in carriers relative to naïve fish by the up-regulation of *CD74* and *CTSS*, which are components of the MHC class II pathway. However, *MHC II* was not differentially expressed in carriers compared to naïve fish. For *CD74,* it has been assumed that it is regulated similarly as *MHC II*, since MHC II molecules are dependent on CD74 as a chaperon to fulfill their role in antigen presentation [71], and since both genes are dependent on the same transcriptional regulator CIITA [72]. However, a recent study found that the *MHC II* and *CD74* mRNA levels do not exhibit a synchronized behavior during maturation of dendritic cells which are one type of APCs [73]. While *CD74* mRNA levels are sustained, *MHC II* expression is down-regulated. Thus in our study, if APCs underwent virus-induced maturation within the brain, down-regulation of *MHC II* would be expected, but this may have been obscured by the infiltration of immature APCs which express high levels of *MHC II* mRNA [74].

The lysosomal cysteine protease *CTSS*, up-regulated in carriers, may be involved in degradation of antigens to present peptides on MHC class II molecules. However, increased expression of *CTSS* as well as *lysozyme* was also associated with neurodegeneration in mammals [75]. Although this characteristic has yet to be demonstrated in fish it is worth to mention that several probes annotated as *lysozyme g* were significantly up-regulated in brains of carriers and survivors compared to naïve fish.

Another key finding of this study was the down-regulation of genes involved in the mevalonate pathway leading to cholesterol biosynthesis in carriers and survivors when compared to naïve individuals. Cholesterol is an essential component in cellular membranes and abundantly found in the CNS where it plays a key role in synapse formation and function [76, 77]. However the cholesterol pathway and its modulation have also been proposed as an important antiviral mechanism. Inhibition of cholesterol biosynthesis and cholesterol-removing agents have been shown to impair the viral lifecycle of various viruses [46]. Moreover the down-regulation of cholesterol biosynthesis genes has been observed in both acute and persistent viral infections and is believed to restrict viral assembly through reduced cholesterol reserves [48, 78]. How viruses trigger cholesterol modulation in a host is uncertain, however Blanc et al. [79] recently proposed that down regulation of cholesterol-metabolic pathway is dependent upon a type I IFN response that is initiated by a viral infection. These authors also noted that the anti-viral effect of down-regulating the sterol pathway was dependent on the availability of mevalonate rather than cholesterol as had been proposed by others. In our study, the down-regulation of genes involved in the mevalonate pathways in both IHNV survivors and carriers suggests that this response is associated with IHNV exposure. Nonetheless whether this altered gene expression measured in survivors and carriers is reflective of an antiviral response is unclear. Notably such mevalonate pathway regulation has not been reported in kidney or spleen tissues of salmonids with acute IHNV infection, despite the strong induction of a type I IFN response [60, 80, 81]. Here, genes related to a type I IFN response were not up-regulated in response to being a survivor/carrier of IHNV thus type I IFN was probably not necessary to down-regulate the mevalonate pathway. Whether such mevalonate modulation would be observed in brain tissue of Sockeye Salmon during the acute stage of infection or whether this response is restricted to IHNV survivors and carriers remains unresolved and requires further investigations.

If down regulation of cholesterol biosynthesis is suggestive of an antiviral mechanism in Sockeye Salmon carriers it is unclear why a similar response is also induced in survivors. One possible explanation is that survivors may still contain IHNV albeit at levels below our detection limit yet sufficient enough to influence cholesterol biosynthesis. Alternatively, the down-regulation of cholesterol biosynthesis in survivors and carriers may not reflect an antiviral mechanism but rather suggest a cellular or metabolic change in response to neuronal damage caused by IHNV infection. Brain has been demonstrated as a tissue tropism for IHNV [57, 82] however more work is required to determine whether neuropathological changes occur due to IHNV infections. With a related fish virus, VHSV, neuronal damage was associated with infection of peripheral nerves in wild Pacific Herring [83]. Moreover a study on the highly neurotropic pathogen, rabies virus, also in the *Rhabdoviridae* family, demonstrated that infection in mice can cause permanent neuronal damage that can persist in the absence of sustained viral antigen and prohibit recovery to a pre-infection phenotype [84]. Whether such sustained impacts would occur from the neuropathogenesis of IHNV in Sockeye Salmon is uncertain, although it is noteworthy that the capacity for neurogenesis and neuronal regeneration is much greater in fish than in mammals [85]. In fact, several differentially expressed probes suggesting functions of regeneration and neurogenesis were observed in IHNV carriers and survivors, along with *forebrain development* revealed through GO enrichment of carrier-relevant probes.

Interestingly, in the comparison of brain expression profiles of IHNV-exposed versus naïve fish there is an indication that IHNV exposure may also have potential effects on host behavior. Among the carrier profiles, the GO category *behavior* was enriched and *EPD* with a potential role in behavior was significantly up-regulated in both survivors and carriers relative to control fish. In zebrafish, the inactivation of EPD resulted in increased aggression and an enhanced competitive ability [86], so it is interesting as to what affect, if any, an increased expression of EPD may exert towards behavior in Sockeye Salmon. Virus induced behavioral changes in fish have been largely unstudied although common carp (*Cyprinus carpio carpio*) infected with cyprinid herpesvirus-3, exhibited a phenomena known as behavioral fever by migrating to warmer water in comparison to non-infected control fish [87].

### Gene expression influenced by poly(I:C)-injection

In an effort to better understand whether IHNV exposure and persistence may alter the capacity of Sockeye Salmon to respond to other viruses, we subjected IHNV carriers, survivors and naïve fish to the viral mimic, poly(I:C). The double-stranded RNA molecule poly(I:C) is well documented as a viral mimic in fish due to its strong induction of a type I IFN response [88–92]. Such an elevated innate response was corroborated in our study where Sockeye Salmon, regardless of their IHNV status, responded to poly(I:C)-injection with the up-regulation of a large number of probes typically found after viral infection or poly(I:C) treatment [32, 54, 60, 93, 94].

Among the list of probes affected by poly(I:C) treatment, fifteen were previously identified as differentially expressed in the survivors and carriers as discussed above. However for the majority of these probes (11 of the 15) their expression was always down-regulated when associated with poly(I:C) treatment while in survivors and/or carriers (not receiving the viral mimic), the probes were always up-regulated. This may be explained by differences in the signaling pathways activated by IHNV status compared to poly(I:C). Due to the ability of the viral mimic to suppress expression of these probes, it is possible that their putative functions as occurring in survivors and carriers may be compromised upon subsequent viral challenge. *Serotransferrin-2* and *EPD* were among the probes identified and suggest that a viral challenge of survivors and carriers may interfere with processes as far reaching as neuronal recovery, behavior, and iron homeostasis [86, 95, 96]. However it is noteworthy that among these 11 probes which were suppressed by the viral mimic, none have been identified as virus responsive genes [54] and are likely not required for the innate antiviral response needed to cope with a subsequent virus challenge.

Of the remaining four probes common to both survivors and carriers treated with and without poly(I:C), an up-regulation of expression was observed regardless of treatment. As these probes responded concordantly across poly(I:C) and survivor/carrier groups it is likely that they are also functioning similarly in these groups. Two of the probes, *PSMB7* and *TAPBP* are thought to play a role in the MHC class I pathway and have been previously shown to be up-regulated after IHNV stimulation [60]. Consequently, it was not unexpected that these probes showed increased expression in poly(I:C)-injected fish relative to non-injected fish and their up-regulation in carriers relative to control fish supports a hypothesis that carriers are likely mounting a response to the detectable IHNV in their brain. Interestingly, IHNV carriers did not elicit an interferon pathway response as was observed for Atlantic cod that were asymptomatic carriers of nodavirus [32], suggesting that these viruses likely differ in strategy by which they persist in the host.

### Genes influenced by poly(I:C) injection and IHNV status interaction

Although both naïve and previously exposed fish seem to mount a characteristic antiviral immune response to poly(I:C), there were 162 probes with a post-poly(I:C) expression level dependent on the IHNV status. These probes were grouped into four clusters based on their expression pattern, the most distinct being cluster I (Fig. 3). This cluster contained genes involved in *defense response* which were more up-regulated in survivors and carriers than they were in naïve fish following poly(I:C) injection.

While poly(I:C) is known to improve the immune response when co-administered with antigens (reviewed in [97]) in our study the delivery of antigen and poly(I:C) occurred several months apart from each other. Therefore, our study might be similar to a study in mice [98] that were treated with poly(I:C) alone or in combination with vaccinia virus vaccines three days after infection with the ectromelia virus (ECTV) that is the causative agent of mousepox. Interestingly, the treatment of poly(I:C) alone resulted in prevention of mortality and reduced viral load in target organs. Furthermore, type I IFN alpha sera levels were much higher in ECTV-infected and poly(I:C)-treated than in poly(I:C)-treated naïve mice. This boosting effect of poly(I:C) in combination with the present ECTV antigens might be similar to our study where the fold change of several virus responsive genes (e.g. *SACS, MX, ZNFX1, STAT1, RTP3, IFI44*) was higher in poly(I:C)-injected survivors and carriers compared to poly(I:C)-injected naïve fish relative to their respective non-injected controls, although the interaction effect for these genes was not significant (p > 0.01). While other genes had a significant interaction effect (e.g. cluster I in Fig. 3), we cannot exclude the possibility that our ability to detect this interaction was limited by the sample size of carriers. However, even with higher statistical power, it will be difficult to exclude the possibility that more Sockeye Salmon with strong antiviral responses survived the initial IHNV challenge than do those with weak responses and subsequently the surviving fish are those with the strongest antiviral response whereas the naïve fish would be a mixture of weak and strong responders.

Finally, the elevated expression after poly(I:C) injection in survivors and carriers may have occurred due to slight differences in cell composition in the brain resulting from previous IHNV infection. Some cell types might respond stronger to poly(I:C) than others, as was shown for murine macrophages and fibroblasts [99]. The present transcriptome analysis suggests that immune responses as well as regeneration and neurogenesis occur in brain of both survivors and carriers. Whether this would change the brain cell make-up in survivor and carriers compared to naïve fish needs further investigations.

While the impact of such IHNV status-dependent elevated expression after poly(I:C)-injection is unclear, it is possible that fish surviving IHNV challenge may have a facilitated response to subsequent viral challenge. Protective effects against IHNV elicited in rainbow trout pre-exposed to avirulent reovirus have been described [100]. Undoubtedly, further testing of this hypothesis would hold the promise to improve our understanding of ecological consequences of surviving a previous exposure to IHNV.

## Conclusions

This is the first transcriptional study of IHNV persistence in Sockeye Salmon. Fish surviving IHNV exposure (survivors, carriers) show distinct transcriptional profiles in brain compared to naïve fish. Whether these differences result in physiological, behavioral and/or ecological consequences requires further investigation. Furthermore, IHNV carriers show signs of an ongoing adaptive immune response. Whether this is related to stage of infection or maintenance of the carrier state is currently unknown. In addition, the down-regulation of genes related to cholesterol biosynthesis suggests an anti-IHNV response previously undescribed in fish that is similar to a response recently identified in higher vertebrates. The IHNV status affected the expression of some genes in response to poly(I:C) injection, most notably by increasing a subset of defense-related transcripts, but overall the characteristic antiviral response was maintained. Thus, we found no evidence that IHNV exposure limits the immune response to other viral antigens (after the acute infection stage). In summary, these data expand upon our understanding of IHNV infections and provide insight into the mechanism by which the virus is maintained in Sockeye Salmon populations.

### Availability of supporting data

Gene expression data files have been uploaded to Gene Expression Omnibus (GSE65241).

## Additional files

Additional file 1: Table S5. Primer sequences for RT-qPCR and sequencing. Additional file 2: Table S1. Complete list of significantly differentially expressed probes that were affected by IHNV status (survivor, carrier), poly(I:C)-injection and an interaction effect of both factors. Additional file 3: Table S2. Fold change (*FC* ≤ 1.5) list and GO enrichment analysis of probes affected by IHNV status. Additional file 4: Table S3. Fold change (*FC* ≤ 1.5) list and GO enrichment analysis of probes affected by poly(I:C)-injection. Additional file 5: Table S4. Fold change (*FC* ≤ 1.5) list and GO enrichment analysis of probes affected by interaction of IHNV status and poly(I:C)-injection. Additional file 6: Figure S1. Correlation between microarray and RT-qPCR log_2_ expression data. A) R^2^ and slopes (RT-qPCR/array) are summarized for all tested genes. Gene names and probe IDs indicate the respective probe used for correlation. Gene acronyms are used according to primer Table S5. B) The correlation for *Mx* is shown as an example.
