# Supplementary material for: Infectious hematopoietic necrosis virus (IHNV) persistence in Sockeye Salmon: influence on brain transcriptome and subsequent response to the viral mimic poly(I:C)
Source: BMC Genomics. 2015 Aug 26;16(1):634. doi: 10.1186/s12864-015-1759-y (PMC4549833; doi:10.1186/s12864-015-1759-y)
Supplement: Additional file 1: Table S5. — Primer sequences for RT-qPCR and sequencing. [file 12864_2015_1759_MOESM1_ESM.docx]

**Table S5. Primer sequence for RT-qPCR and sequencing.**

| **Target gene** | **Primer sequence 5’ to 3’** | **Amplicon length** | **Efficiency** |
| --- | --- | --- | --- |
| U6 snRNA-associated Sm-like protein (LSm8) [40] | U6 fwd TTGACCAGACCATCAACCTG  U6 rev CAGCAACGTTGTCTCCTCTG | 117 | 2.03 |
| Dynein light chain 1, cytoplasmic (DYN) [40] | Dyn fwd ACATCGAGAAAGACATCGCC  Dyn rev TCTCATGGGTCACGTAGCTG | 111 | 2.08 |
| Immunoglobulin µ heavy chain, secreted (Hcmu sec) [56] | Hcmu sec fwd GCG CTG TAG ATC ACA TGG AA  Hcmu sec rev GCA AGT CAG GGT CAC CGT AT | 143 | 2.1 |
| Immunoglobulin µ heavy chain, membrane-bound (Hcmu memb) [56] | Hcmu memb fwd GCG CTG TAG ATC ACA TGG AA  Hcmu memb rev TTT CAC CTT GAT GGC AGT TG | 208 | 2.31 |
| Interferon-induced GTP-binding protein Mx (Mx) | Mx fwd GGT TGT GCC ATG CAA CGT T  Mx rev GGC TTG GTC AGG ATG CCT AAT | 102 | 2.03 |
| Galectin-3-bindingprotein precursor (LGALS3BP) | LGALS3BP fwd ATTGATCAGAGCCTCGCAGT  LGALS3BP rev AGTTTGGGGTGAAACGTCTG | 179 | 2.01 |
| Complement C1s subcomponent precursor (C1S) | C1S fwd CGTTCCGCTCTGACTACTCC  C1S rev GCGACAGGAGCAGAGGTAT | 151 | 1.95 |
| Farnesyl pyrophosphate synthetase (FDPS) | FDPS fwd GACGTCCTTCCAGACAGAGC  FDPS rev TTCCAATCTTCCCTGTCACC | 293 | 1.94 |
| Squalene monooxygenase (SQLE) | SQLE fwd CTTGTCATTGGGCAGACCTT  SQLE rev CACACCCCTAGAGGCAAAAG | 171 | 1.98 |
| Ependymin-2 precursor (EPD2) | EPD2 fwd TTAACGCAGAATACTGGCCC  EPD2 rev CCCATCAGGTTGAGCTGTTT | 111 | 2.07 |
| CCAAT/enhancer-binding protein delta (CEBPD) | CEBPD fwd CTGGAACTCTGCAACGATGA  CEBPD rev AATCCACCCTTGAGCTTCCT | 190 | 2.03 |
| Complement component C7 precursor (C7) | C7 fwd ATGACAAACCACCACCCAAT  C7 rev GCAGCCTGTAGAGGACCTTG | 280 | 2.02 |
| Beta-1,3-glucosyltransferase (B3GALTL) | B3GALTL fwd TACGCCTTTGCAGAGAACC  B3GALTL rev CTTGGCCATTCTCCCAGATA | 180 | 2.02 |
| Receptor-transporting protein 3 (RTP3) | RTP3 fwd CTTGTCTTCCAACAGACGCA  RTP3 rev CAGAACACGATTGAGGAGCA | 201 | 1.93 |
| Sacsin (SACS) | Sacs fwd GCAGAGGAGAAACACAAGGC  Sacs rev TGGAGAACCTCTCTCTCCCA | 241 | 1.97 |
