# Supplementary material for: Infectious hematopoietic necrosis virus (IHNV) persistence in Sockeye Salmon: influence on brain transcriptome and subsequent response to the viral mimic poly(I:C)
Source: BMC Genomics. 2015 Aug 26;16(1):634. doi: 10.1186/s12864-015-1759-y (PMC4549833; doi:10.1186/s12864-015-1759-y)
Supplement: Additional file 6: Figure S1. — Correlation between microarray and RT-qPCR log2 expression data. A) R2 and slopes (RT-qPCR/array) are summarized for all tested genes. Gene names and probe IDs indicate the respective probe used for correlation. Gene acronyms are used according to primer Table S5. B) The correlation for Mx is shown as an example. [file 12864_2015_1759_MOESM6_ESM.pptx]

## Slide 1
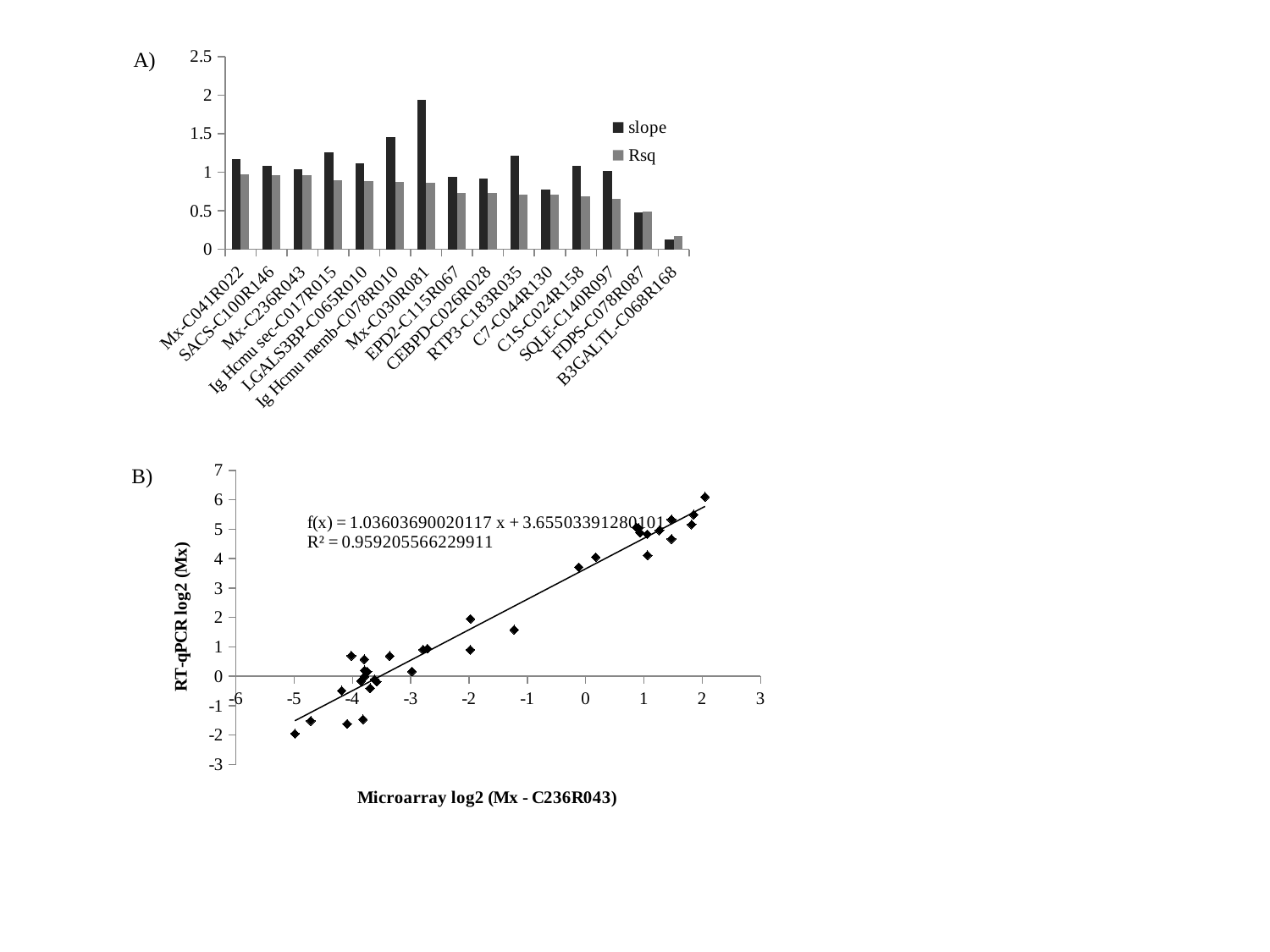

### Chart
| Category | | Rsq |
|---|---|---|
| Mx-C041R022 | 1.1729 | 0.9747 |
| SACS-C100R146 | 1.0867 | 0.9654 |
| Mx-C236R043 | 1.036 | 0.9592 |
| Ig Hcmu sec-C017R015 | 1.2553 | 0.8981 |
| LGALS3BP-C065R010 | 1.1192 | 0.8803 |
| Ig Hcmu memb-C078R010 | 1.4539 | 0.8731 |
| Mx-C030R081 | 1.9367 | 0.857 |
| EPD2-C115R067 | 0.941 | 0.7356 |
| CEBPD-C026R028 | 0.9124 | 0.7352 |
| RTP3-C183R035 | 1.2166 | 0.7137 |
| C7-C044R130 | 0.7768 | 0.7073 |
| C1S-C024R158 | 1.0817 | 0.6837 |
| SQLE-C140R097 | 1.0191 | 0.6533 |
| FDPS-C078R087 | 0.4737 | 0.488 |
| B3GALTL-C068R168 | 0.1267 | 0.1682 |A)
### Chart
| Category | |
|---|---|B)
